# 247. Evaluation of Self-Contamination Risks in Healthcare Personnel Wearing Standard Versus Extended Use Personal Protective Equipment (Extend PPE Study)

**DOI:** 10.1093/ofid/ofaf695.090

**Published:** 2026-01-11

**Authors:** Caroline O’Neil, Lucy Vogt, Kate Peacock, Carol Muenks, Olivia G Arter, Regine Burton, Maxfield J Steger, Lauren Hunstad, Megan L Porter, Elianora Liana Ovchiyan, Kim Vu, David McDonald, Carleigh Samuels, Abby Sung, Stephen Y Liang, David Kuhar, Melanie L Yarbrough, Jennie H Kwon

**Affiliations:** Washington University, St. Louis, Missouri; Washington University, St. Louis, Missouri; Washington University in St. Louis, St. Louis, Missouri; Washington University School of Medicine, Saint Louis, MO; Washington University in St. Louis, St. Louis, Missouri; Washington University School of Medicine, Saint Louis, MO; Washington University, St. Louis, Missouri; Washington University in St. Louis, St. Louis, Missouri; Washington University School of Medicine, Saint Louis, MO; Washington University in St.Louis School of Medicine, St.Louis, Missouri; Washington University in St. Louis School of Medicine, Kansas City, Kansas; Washington University, St. Louis, Missouri; Washington University - St. Louis, Greensboro, North Carolina; Washington University School of Medicine in St. Louis, Saint Louis, Missouri; Washington University School of Medicine, Saint Louis, MO; Centers fo Disease Control and Prevention; Washington University School of Medicine in St. Louis, Saint Louis, Missouri; Northwestern University , Chicago , IL

## Abstract

**Background:**

Supply shortages during the COVID-19 pandemic forced many healthcare personnel (HCP) to wear personal protective equipment (PPE) for extended periods of time, raising concerns that extended use or re-use of PPE might increase risk of self-contamination and contamination of the hospital environment. The objective of this study was to compare and quantify contamination risks of different PPE use strategies.

**Table 1.**
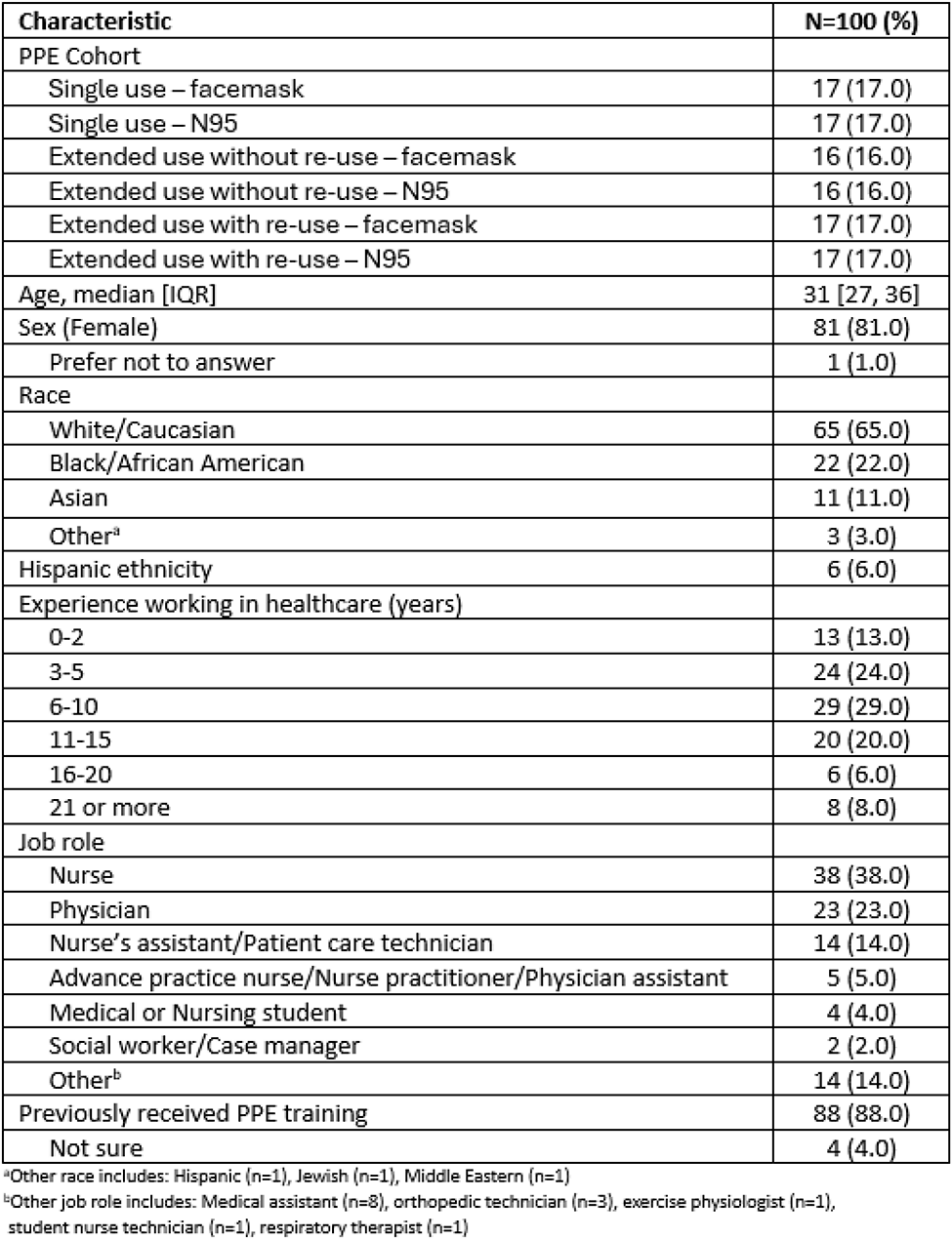
Characteristics of participating healthcare personnel, recruited at an acute care hospital between 12/16/2022 and 3/26/2025.

**Figure 1.**
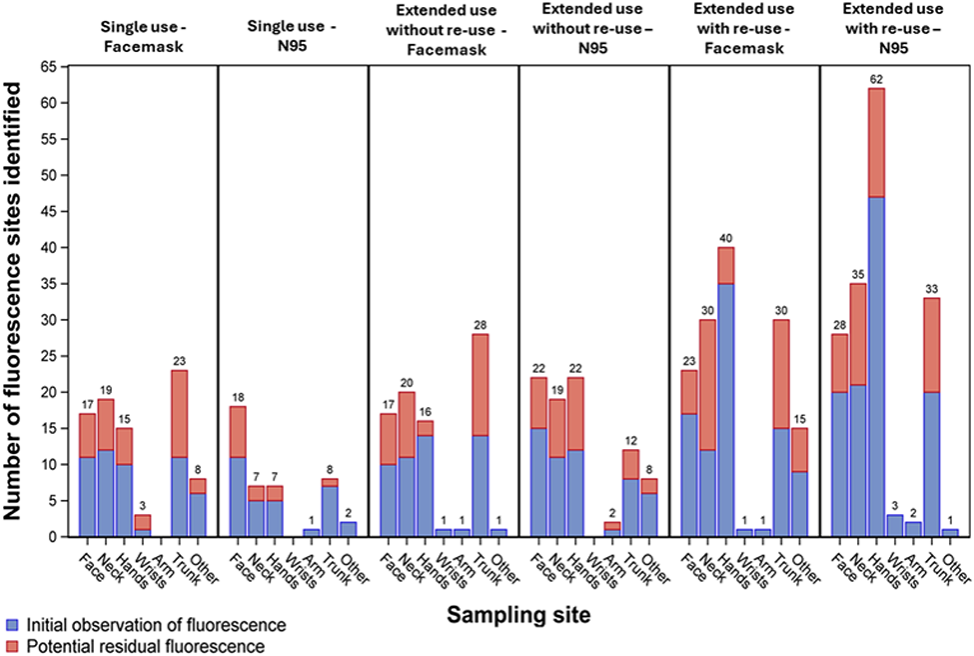
Frequency of fluorescent marker identification by sampling site and PPE use cohort, showing initial observation of fluorescence (blue) and continued observation of fluorescence at subsequent time points (red).

**Methods:**

Glo Germ Fluorescent marker (FM) and MS2 bacteriophage (MS2), surrogates for pathogen transmission, were applied to surgical face masks/N95 respirators and goggles/face shields. We recruited 100 HCP at an acute care hospital to practice single use, extended use without re-use, or extended use with re-use, using the marked face mask or N95 and eye protection, over a 4-hour observation period while providing clinical care. At hourly intervals, a UV black light was used to detect FM on HCP, and swabs were collected to detect MS2 via PCR. At the final time point, the surrounding hospital environment was also assessed for FM or MS2 presence. The type and extent of HCP self-contamination and environmental contamination were compared across all PPE cohorts.

**Figure 2.**
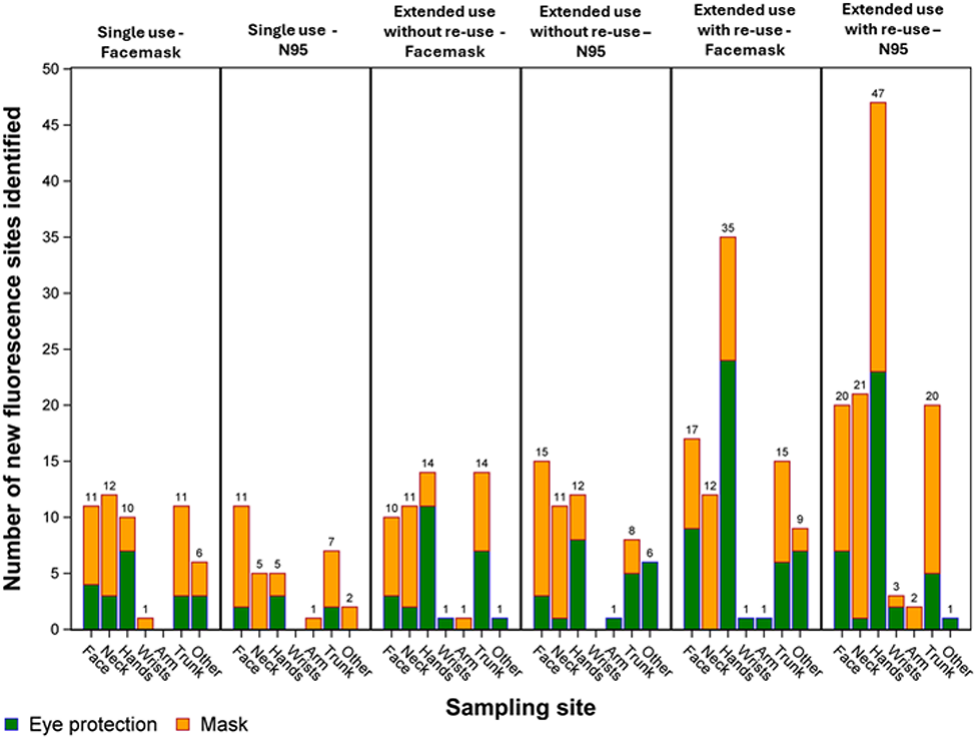
Frequency of fluorescent marker identification by sampling site and PPE use cohort, showing PPE contamination from masks (orange) versus eye protection (green).

**Figure 3.**
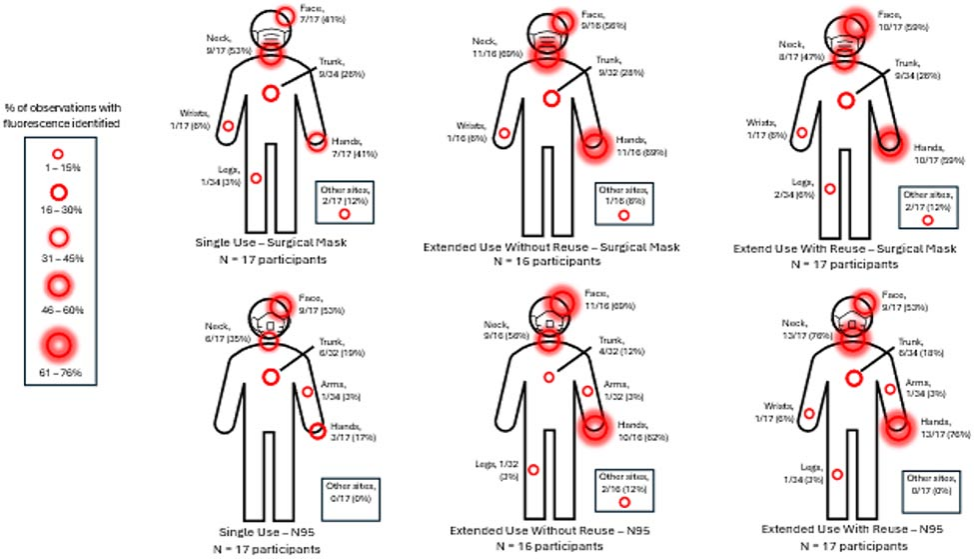
Percentage of samples with fluorescence identified at the first sampling time point by cohort.

**Results:**

Increased FM self-contamination was observed on HCP assigned to extended PPE use strategies, particularly extended use with re-use, as compared to a single use PPE strategy (Figure 1). Transfer of FM from masks was more frequent than transfer from eye protection (Figure 2). HCW self-contamination was more common than contamination of the hospital environment. MS2 detection decreased over the course of the observation period (76% at hour 1 versus 28% at hour 4) and was higher for extended use with re-use (87% for mask, 87% for N95) and extended use without re-use (67% mask, 80% N95) versus the single use PPE cohorts (50% for mask, 67% for N95). Common sites for both FM and MS2 self-contamination were the hands, neck, and face. Downstream environmental contamination was observed for 7 HCP.

**Conclusion:**

Single-use PPE strategies were associated with less contamination than extended-use strategies. Further research into the use of single-use vs. extended-use PPE strategies is needed to better understand the risks of each strategy and to inform hospital infection prevention practices.

**Disclosures:**

All Authors: No reported disclosures

